# Acquisition versus Consolidation of Auditory Perceptual Learning Using Mixed-Training Regimens

**DOI:** 10.1371/journal.pone.0121953

**Published:** 2015-03-24

**Authors:** David W. Maidment, HiJee Kang, Emma C. Gill, Sygal Amitay

**Affiliations:** Medical Research Council Institute of Hearing Research, Nottingham, United Kingdom; Baycrest Hospital, CANADA

## Abstract

Learning is considered to consist of two distinct phases–acquisition and consolidation. Acquisition can be disrupted when short periods of training on more than one task are interleaved, whereas consolidation can be disrupted when a second task is trained after the first has been initiated. Here we investigated the conditions governing the disruption to acquisition and consolidation during mixed-training regimens in which primary and secondary amplitude modulation tasks were either interleaved or presented consecutively. The secondary task differed from the primary task in either task-irrelevant (carrier frequency) or task-relevant (modulation rate) stimulus features while requiring the same perceptual judgment (amplitude modulation depth discrimination), or shared both irrelevant and relevant features but required a different judgment (amplitude modulation rate discrimination). Based on previous literature we predicted that acquisition would be disrupted by varying the task-relevant stimulus feature during training (stimulus interference), and that consolidation would be disrupted by varying the perceptual judgment required (task interference). We found that varying the task-relevant or -irrelevant stimulus features failed to disrupt acquisition but did disrupt consolidation, whereas mixing two tasks requiring a different perceptual judgment but sharing the same stimulus features disrupted both acquisition and consolidation. Thus, a distinction between acquisition and consolidation phases of perceptual learning cannot simply be attributed to (task-relevant) stimulus versus task interference. We propose instead that disruption occurs during acquisition when mixing two tasks requiring a perceptual judgment based on different cues, whereas consolidation is always disrupted regardless of whether different stimulus features or tasks are mixed. The current study not only provides a novel insight into the underlying mechanisms of perceptual learning, but also has practical implications for the optimal design and delivery of training programs that aim to remediate perceptual difficulties.

## Introduction

Perceptual learning refers to the process whereby sensory processing is improved through experience or following training on a task [1]. In the natural environment, perceptual learning occurs despite the constantly changing stream of incoming sensory information and behavioral demands [2,3]. Studies in the laboratory attempt to artificially simulate this ‘real-world’ experience, exploring interactions between tasks in mixed-design training regimens. Mixed-training paradigms have shown that learning on a particular task can either be facilitated or disrupted depending on other tasks that precede or follow it (e.g. [4–11]). Nevertheless, there is a lack of consensus as to when learning something new affects what has already been learned (retrograde interference). In order to address this, the current study systematically investigated the effects of inter-task interactions on perceptual learning. Understanding how tasks interact during multi-task training has important theoretical and practical implications, improving our understanding of the underlying mechanisms of learning, as well as informing the optimal design and delivery of applied training programs that aim to improve perceptual abilities.

Learning is conceived as consisting of two putative phases: (1) *acquisition*, the period of active practice involving transient, short-term representations or memory traces, and (2) *consolidation*, the transformation of learning into a more robust, long-term form [10,12–14]. The conditions under which acquisition and consolidation take place have typically been investigated by examining how learning something new impacts the process of storing and retrieving pre-existing information (for review, see [15]). For example, in perceptual learning the functional distinction between these stages has typically been investigated by examining whether training-induced improvements on a primary task are differentially affected by subsequent events, such as training on a secondary task, when presented before (during acquisition) or after consolidation of the primary task [4–9,11]. Although a clear pattern of interference is difficult to discern in the visual perceptual learning literature, evidence in the auditory domain suggests that acquisition and consolidation phases of learning are differentially affected depending on whether the primary and secondary tasks differ in task demands or stimulus features, respectively.

**Fig 1:**
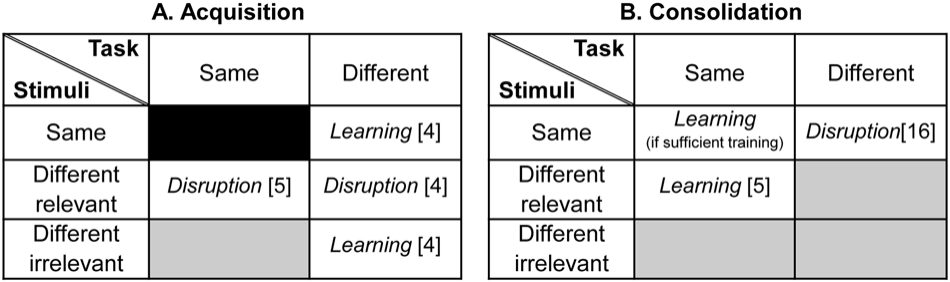
Acquisition and consolidation based on the auditory learning literature. The pattern of learning and disruption observed during (A) acquisition and (B) consolidation, when varying tasks and/or stimulus features (relevant/irrelevant) during training. Grey filled boxes denote that this combination of task/stimulus features has not been previously investigated using a mixed training paradigm.

Acquisition of a temporal interval duration discrimination task (two tone pips separated by a silent interval) was disrupted when it was interleaved in short blocks by training on the same task with a different duration standard stimulus [5]. Likewise, acquisition of a frequency discrimination task (using the same two tone pips stimuli) was disrupted when it was interleaved with another frequency discrimination task using different-frequency standards [4]. In both cases, mixing training on stimuli that differed in the task-relevant feature (duration in temporal interval discrimination and frequency in frequency discrimination) resulted in disruption to acquisition (Fig. 1A). However, interleaving two frequency discrimination tasks that differed in the duration between the pips—a task-irrelevant stimulus feature—did not disrupt acquisition [4]. Neither was it disrupted when the frequency discrimination task was interleaved with a temporal interval discrimination task that shared the same standard stimulus [4]. Interference during the acquisition phase therefore appears to only occur when changing the task-relevant stimulus feature.

A different pattern emerges when training was mixed during the consolidation phase: Consolidation of a primary temporal interval discrimination task was disrupted when it was consecutively followed by a secondary frequency discrimination task using the same standard stimuli [16]. This was contingent on a sufficient number of trials for consolidation of the primary task to be initiated [17] (Fig. 1B). On the other hand, consolidation of a temporal interval discrimination primary task was not disrupted when it was followed by a secondary temporal interval discrimination task with stimuli of a different duration [5]. Interference during the consolidation phase therefore appears to occur as a result of mixing different tasks using the same stimuli but requiring perceptual judgments based on different cues (e.g. duration versus frequency).

The objective of the current study was to test whether the distinction between acquisition and consolidation phases of learning can be attributed to stimulus versus task interference. Specifically, we investigated the extent to which interleaved (acquisition) and consecutive (consolidation) regimens disrupted learning of a primary, auditory amplitude modulation depth discrimination task (AMD) when training was mixed with either the same AMD task with different task-relevant or -irrelevant stimulus features, or an amplitude modulation rate discrimination task (AMR) with the same task-relevant and -irrelevant stimulus features. Using amplitude-modulated tone stimuli during training allows us to independently vary all three features that affect learning: task-relevant and task-irrelevant stimulus features (modulation rate and carrier frequency, respectively) within the same task, as well as the perceptual judgment based on different cues (modulation depth versus rate) whilst keeping the stimulus features constant.

Based on the hypothesis that acquisition is disrupted as a result of task-relevant stimulus interference, we predicted that learning of the primary AMD task will only be disrupted by interleaved training on the same AMD condition that differs in terms of the task-relevant (i.e. modulation rate) stimulus feature. Learning should still be observed if the primary task is mixed with another AMD task that differs in terms of the task-irrelevant (carrier frequency) stimulus feature or a different task (AMR) with the same stimuli. If consolidation is disrupted as a result of mixing different tasks, we predicted that learning of the primary AMD task will be disrupted by consecutive training on a secondary AMR task. Learning of the AMD task should not be disrupted by training on an AMD task that differs in terms of task-relevant or -irrelevant stimulus features.

## Methods

### Ethics statement

The research protocol was approved by the Nottingham University Hospitals NHS Trust Research Ethics Committee and was conducted according to the principles expressed in the Declaration of Helsinki. Informed written consent was obtained from all participants.

### Participants

One-hundred and twenty adults (78 females, 42 males) aged 18–39 years old (mean = 23.2 years; SD = 3.5 years) were recruited via posters from the University of Nottingham student population and the general public, and paid an inconvenience allowance for their participation. No participants reported current or past difficulties with hearing, with screening confirming that audiometric pure-tone thresholds were ≤ 20 dB HL across 0.125 to 8 kHz octaves (administered in a double-walled sound-attenuating booth using Telephonics TDH49 audiometric earphones, in accordance with the recommended procedure of the British Society of Audiology [18]). All participants had no prior experience of psychoacoustic testing. To ensure naïve thresholds were homogeneous across groups (as amount of learning is often related to starting thresholds [19]), participants were assigned to one of eight experimental groups, matched on the pre-test thresholds in the primary AMD task (see below). The Levene’s test confirmed that the variances across all groups on this task were equal, *F*(7,111) = .46, *p* = .86. The independent samples Kruskal-Wallis test also confirmed that the distributions of each group did not significantly differ (*p* = .82).

### General protocol

**Fig 2:**
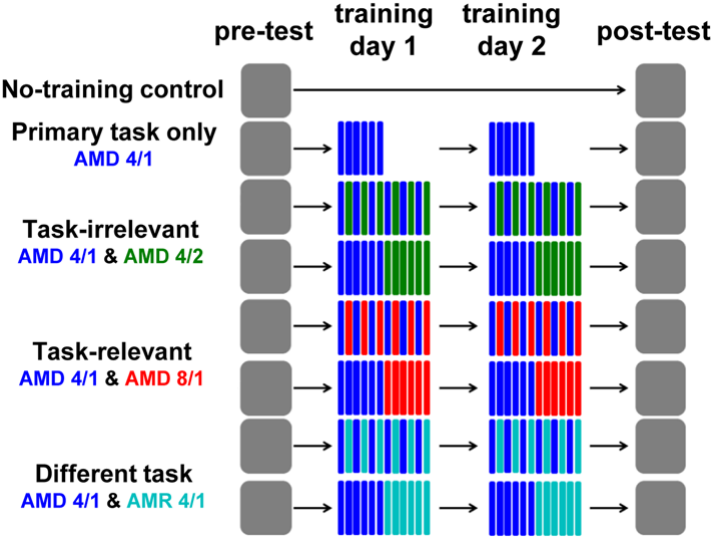
A schematic representation of the training regimens. Pre- and post-tests (days 1 and 4, respectively) were completed across all groups. For the no-training control group, these sessions were separated by a two day interval. All remaining groups completed training within this period (days 2 and 3). For the primary-task only group, training consisted of 360 trials of the AMD 4/1 task. Mixed-training groups also trained on a secondary task (task-irrelevant, AMD 4/2; task relevant, AMD 8/1; different task, AMR 4/1) for 360 trials, which was either interleaved (60 trial blocks) with or presented consecutively (360 trial blocks) after training on the primary task.

All groups took part in a pre-test and post-test, which were separated by three days (Fig. 2). During the pre- and post-tests all listeners were tested on a variety of AMD and AMR tasks (described below). Between pre- and post-test, a subset of the listeners (n = 105) completed one of seven different training regimens consisting of two daily sessions administered on consecutive days. The remaining listeners (n = 15) acted as no-training controls, and attended only the pre- and post-test sessions.

### Equipment

Stimuli were presented diotically at 70 dB SPL using Sennheiser HD-25-1 headphones. All testing was completed within a double-walled sound-attenuating booth. Tasks were administered via custom software written in MATLAB Release 2010b (The MathWorks, Inc., Natick, Massachusetts, United States). Responses were recorded via a custom-made button box placed horizontally in front of the participant. There was no time limit in which to respond and participants were provided trial-by-trial visual feedback presented on a NEC touchscreen LCD computer monitor placed in front of them at a comfortable viewing distance.

### Tasks and adaptive procedure

For both AMD and AMR tasks, brief tones were presented in each of three observation intervals in a three alternative forced choice (3I-3AFC) oddball design, and participants were instructed to choose the interval that was different from the other two. The interval containing the different tone was randomly determined.

During each AMD trial, the two standard intervals contained an unmodulated tone and the third (target) interval contained an amplitude modulated tone of the same frequency. In comparison to previous studies of amplitude modulation (AM) learning, which have used faster modulation rates that elicit a sensation of pitch [20–22], in the current study slower modulation rates akin to those experienced during the perception of speech were presented.

The modulation depth (m) was adaptively varied, starting with 100% modulation (m = 0 dB), and reduced by a factor of 1.26 (2 dB) when the modulation rate was 4 Hz and 1.58 (4 dB) when the modulation rates were 8 and 16 Hz, until the first incorrect response.

During AMR trials, two intervals contained a modulated tone at a base modulation rate (r), and the third interval shared the same carrier tone frequency but had a higher modulation rate (r + Δr, where Δr is a percentage of r). The value of Δr was adaptively varied, starting with Δr = 150% (e.g. r = 4 Hz, Δr = 10 Hz), and was reduced by a factor of √1.4 when r was 4 Hz and 1.4 when r was ≥ 8 Hz until the first error.

Following the first incorrect response in either task, the procedure governing the change of both m and Δr reverted to a three-down one-up staircase procedure, targeting 79.4% correct on the psychometric function [23]. Following three correct responses, m was divided by 1.26 (2 dB) and Δr by √1.4. After one incorrect response, m was multiplied by 1.26 and Δr by √1.4. Each adaptive track was terminated after 30 trials had elapsed for all pre/post-tests, and after 60 trials had elapsed during training.

A demonstration of five trials was administered before the first test block for each task to familiarize participants with the task requirements. Three of these trials were ‘easy’ (AMD: m = 100%; AMR: Δr = 150%), and two were ‘impossible’ (m and Δr = 0%). All participants were required to correctly identify all of the target sounds for the ‘easy’ practice trials before progressing to the test phase.

### Stimuli

For the AMD and AMR tasks, stimuli consisted of 500-ms tones (including 10-ms raised cosine ramps) presented with an inter-stimulus interval of 650 ms. AM-stimuli were created by multiplying 1 kHz pure tones with DC-shifted sinusoids of the required modulation rate. The amplitude of all AM-tones were reduced by [1 + m^2^/2]^0.5^ to cancel out the increase in power resulting from amplitude modulation to avoid listeners basing their decision on intensity cues. Within every trial the starting phase of modulation was randomized for each interval in order to prevent listeners from basing their decisions on the number of modulation cycles.

### Training regimens

Trained listeners participated in one of seven training regimens (Fig. 2). The primary task only regimen (n = 15) consisted of 360 daily trials of the primary task—AMD of a 4 Hz modulation of 1 kHz carrier tone (henceforth referred to as AMD 4/1). To investigate acquisition, training on the primary task was interleaved in 60 trial blocks with a secondary task, such that each task was trained for 360 trials (720 trials in total) per session. In the secondary task-irrelevant regimen (n = 15), the secondary task consisted of AMD with a different carrier frequency (2 kHz) but the same modulation rate (4 Hz) as the primary task, referred to as AMD 4/2. In the secondary task-relevant stimulus regimen (n = 15), the secondary task consisted of AMD with the same carrier frequency (1 kHz) but a different modulation rate (8 Hz)—AMD 8/1. In the secondary different task regimen (n = 15), the secondary task consisted of AMR with the standard stimuli sharing the same carrier frequency and modulation rate as the primary task target (hence AMR 4/1). To investigate consolidation, three further groups (n = 15 each) were trained using a regimen where 360 trials of the primary task were followed consecutively by 360 trials of one of the secondary tasks (AMD 4/2, AMD 8/1, AMR 4/1).

### Pre- and post-tests

**Fig 3:**
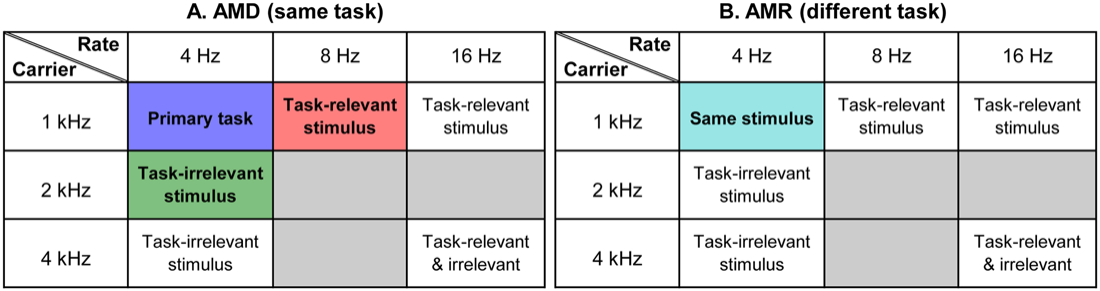
Tasks tested in the current study. All combinations of modulation rate (4, 8, and 16 Hz) and carrier frequency (1, 2, and 4 kHz) presented during the pre- and post-tests for both (A) amplitude modulation depth discrimination (AMD) and (B) amplitude modulation rate discrimination (AMR). Color filled boxes indicate whether this task was also trained: AMD 4/1, primary task-only (blue); AMD 4/2, secondary task-irrelevant (green); AMD 8/1, secondary task-relevant (red); AMR 4/1, secondary different task (cyan). This color scheme is used to denote these training groups in Figs. 4–6, as well as S1 and S2 Figs. Grey filled boxes signify that this combination of rate/carrier was not tested.

In the pre- and post-tests, all participants completed two threshold estimates, consisting of 30 trials each, in six AMD and six AMR conditions (see Fig. 3). The order of presentation was randomized across participants (though the two thresholds of the same condition were always consecutive), but fixed between pre- and post-tests within participants. These conditions assessed learning on the primary (AMD 4/1) and secondary tasks that varied on task-irrelevant (AMD 4/2) or task-relevant (AMD 8/1) stimulus dimension, or on a different task (AMR 4/1). The remaining conditions assessed transfer of learning to untrained conditions: the same task with different task-irrelevant stimulus (AMD 4/4), different task-relevant stimulus (AMD 16/1), or both (AMD 16/4). Transfer to all the above carrier frequency and modulation rate combinations were also tested for AMR-trained stimuli (AMR 4/2, AMR 8/1) and -untrained stimuli (AMR 4/4, AMR 16/1, AMR 16/4).

### Statistical analysis

All AMD and AMR raw trial data were log-transformed, since both tasks were measured in logarithmic units—dB (modulation depth) for AMD and Hz (modulation rate) for AMR. The log transformation ensures thresholds are not overestimated. Data from each adaptive track (block of 30 or 60 trials, from testing and training blocks, respectively) were fitted with a logistic psychometric function using the Psignifit toolbox [24], and the thresholds were estimated as the 79.4% correct point on this function. Tracks where the optimization procedure did not adequately fit the data (i.e. when the fitted slope was negative or when the fitted threshold value was outside the measured range, see [25]) were discarded. Further outlying data were excluded on an individual threshold estimate basis if pre-test thresholds were > 2 standard deviations difference from the population mean or post-test thresholds were > 2 standard deviations from the group mean. If two estimates were viable they were averaged to calculate the threshold per condition; otherwise the remaining viable estimate was taken as the threshold. Both estimates were rejected in only 4 cases (0.13% of all pre- or post-test data points).

The effects of each training regimen on learning of the primary and secondary tasks were examined by comparing the learning curves of each training group to the no-training controls. One-way ANOVAs were used to compare the slope of the learning curve for each trained group to the no-training controls, taking into account the logarithmic nature of the learning curve [26]. The *p*-values were Holm-Bonferroni corrected for multiple comparisons (we report the corrected α where applicable).

The pre-test thresholds combined across all groups were positively correlated with post-test thresholds for every task (*r* ≥. 51, *p* <. 001). Consequently, transfer to untrained conditions was assessed using separate ANCOVAs to adjust the post-test thresholds of each group, using pre-test thresholds as the covariate.

## Results

### Learning on the Primary Task (AMD 4/1)

#### Primary task only

**Fig 4:**
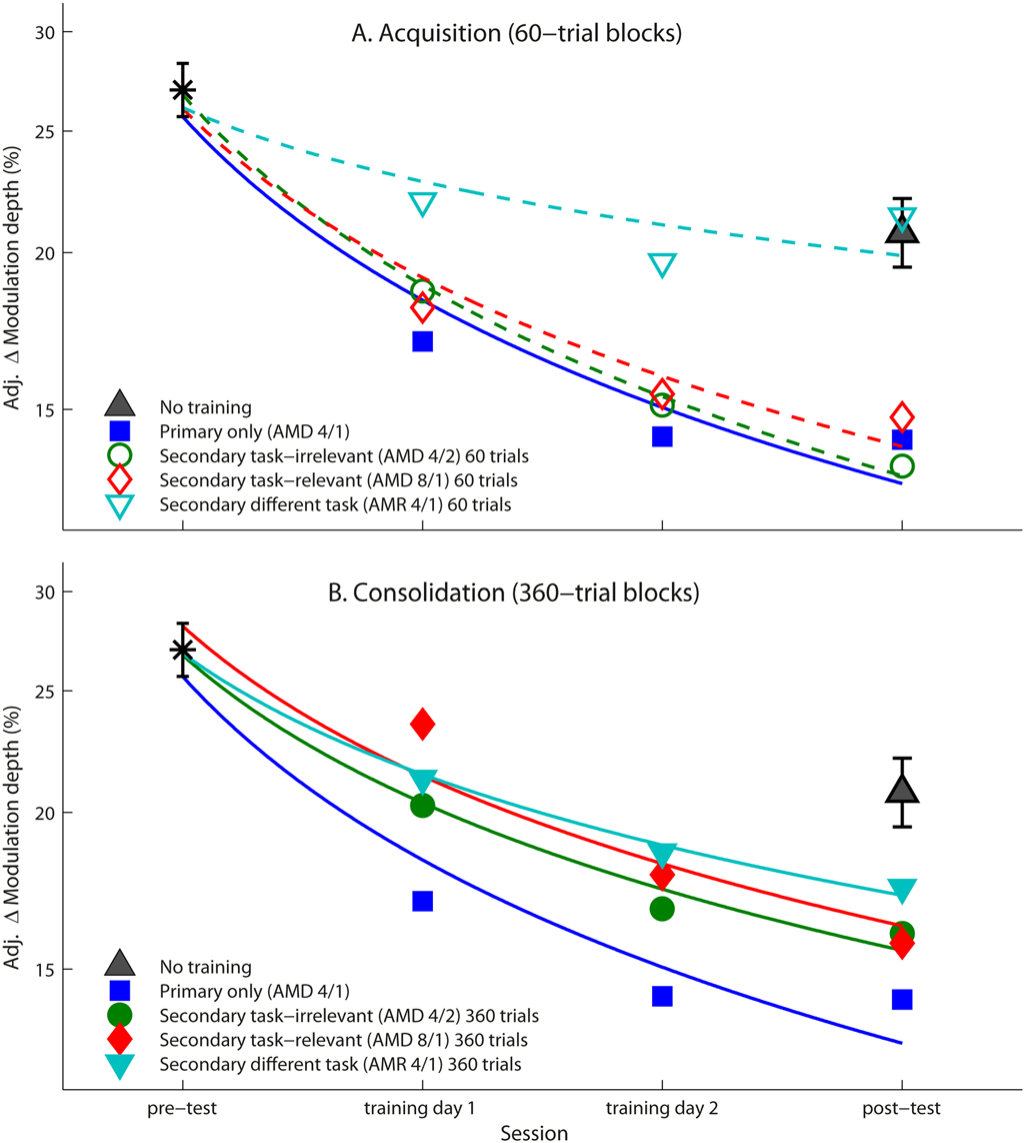
Performance on the primary (AMD 4/1) task. Mean thresholds across the 6 blocks comprising each daily session for primary task-only (blue squares), secondary task-irrelevant (green circles), secondary task-relevant (red diamonds), and secondary different task cue (cyan triangles) training groups during (A) acquisition (unfilled markers) and (B) consolidation (filled markers). Here (and in all subsequent figures) displayed thresholds were adjusted to account for individual variation in pre-test thresholds (on each task), equivalent to using the pre-test thresholds as covariates in an ANCOVA of the post-test thresholds (see [27,28]). The star marks the mean pre-test threshold calculated across the entire sample. Regression lines (dashed for acquisition and continuous for consolidation) were fitted to the log of the session number. The adjusted post-test threshold of the no-training controls is provided as a grey filled triangle. Error bars (here and in subsequent figures) indicate ±1 standard error of the mean.

We first confirmed that 360 trials of the primary AMD 4/1 task (Figs. 2 and 3A, blue box) were sufficient to initiate consolidation of learning by comparing the slope of the learning curves of the primary only training group to that of the no-training controls (Fig. 4). The learning curve of the trained group significantly differed from untrained controls, *F*(1,27) = 11.36, *p* = .002, *η* ^*2*^_*ρ*_ = .30 (α = .007).

#### Mixed training regimens: Acquisition (interleaved 60-trial blocks)

We tested our prediction that mixing training conditions during the acquisition phase would disrupt learning when the secondary task shared task-relevant stimulus features with the primary task. Acquisition of the primary AMD 4/1 task was not disrupted when it was interleaved with a secondary task sharing the same perceptual judgment (AMD) regardless of whether the stimulus differed on the task-irrelevant (carrier frequency: Fig. 3A, green box) or task-relevant feature (modulation frequency: Fig. 3A, red box), but was disrupted when interleaved with a secondary task requiring a judgment based on a different cue (modulation rate: Fig. 3B, cyan box) that shared the same stimulus features (Fig. 4A). The slope of the regression line significantly differed from controls for both the secondary task-irrelevant (AMD 4/2), *F*(1,27) = 11.27, *p* = .002, *η* ^*2*^_*ρ*_ = .30 (α = .008), and task-relevant (AMD 8/1) training groups, *F*(1,28) = 9.84, *p* = .004, *η* ^*2*^_*ρ*_ = .26 (α = .010). The learning curve of the secondary different task group (AMR 4/1) did not differ significantly from no-training controls, *F*(1,28) = .002, *p* = .96, *η* ^*2*^_*ρ*_ <. 001 (α = .05). These findings refuted our prediction based on previous literature (Fig. 1A).

#### Mixed training regimens: Consolidation (consecutive 360-trial blocks)

We also tested our prediction that mixing training conditions during the consolidation phase would disrupt learning when the secondary task was different to the primary, but shared the same stimuli. Consolidation of the primary AMD 4/1 task was partially disrupted when it was followed by a secondary task, regardless of whether it differed on stimulus or required perceptual judgment (Fig. 4B). Although learning was still significant, when corrected for multiple comparisons learning curves no longer differed significantly from the no-training controls for any mixed-training group (secondary task-irrelevant: *F*(1,27) = 4.47, *p* = .044, *η* ^*2*^_*ρ*_ = .14, α = .013; secondary task-relevant, *F*(1,27) = 3.93, *p* = .058, *η* ^*2*^_*ρ*_ = .13, α = .017; secondary different task: *F*(1,27) = 1.85, *p* = .19, *η* ^*2*^_*ρ*_ = .06, α = .025). Thus, disruption to consolidation was not specific to task interference as predicted; it was also disrupted to some extent as a result of stimulus interference (Fig. 1B).

### Secondary task learning

**Fig 5:**
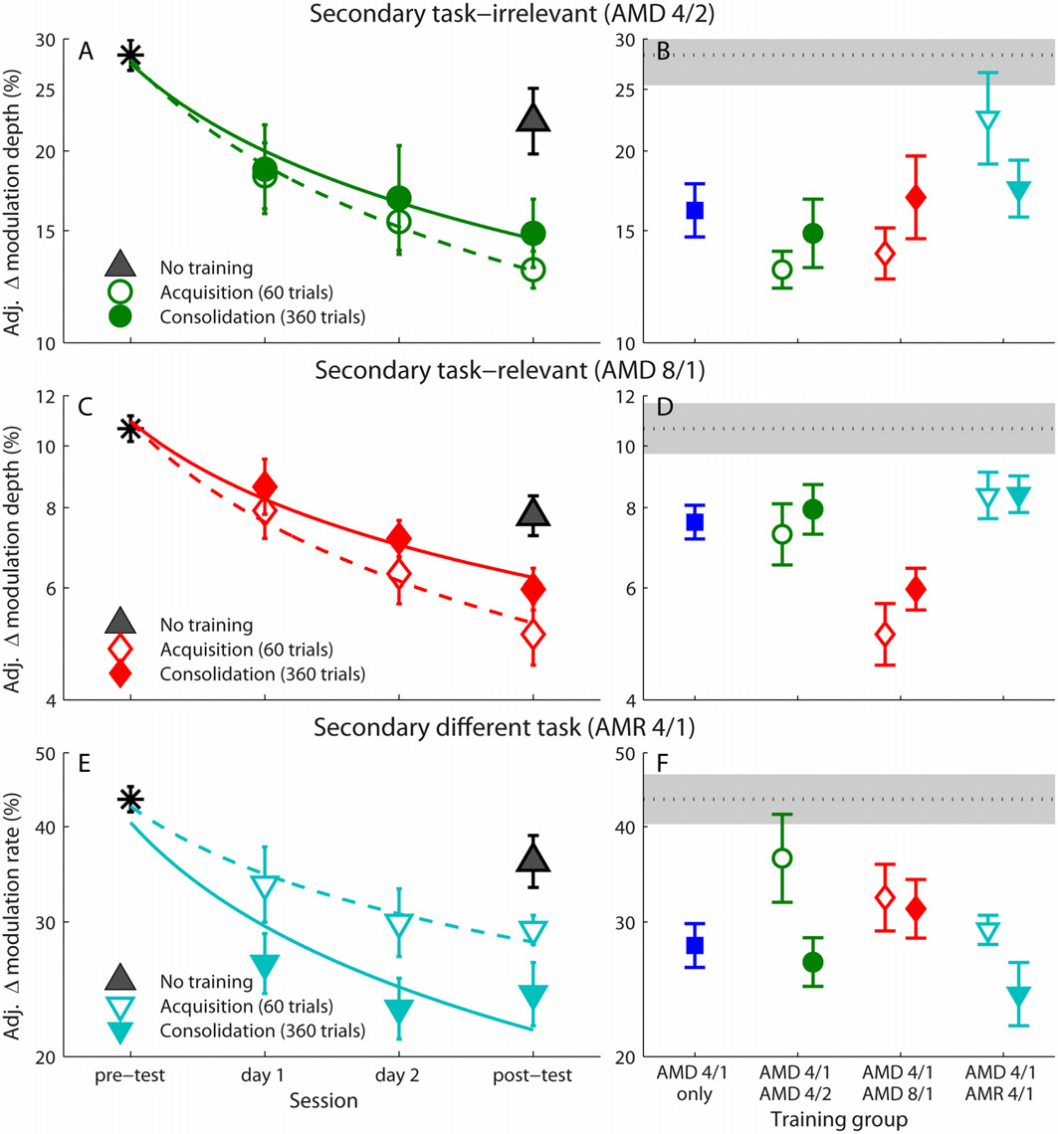
Learning and transfer on the secondary tasks. (A) Mean adjusted thresholds on the AMD 4/2 task across each daily session for the secondary task-irrelevant training group during acquisition and consolidation. Regression lines were fitted to the log of the session number. (B) Mean adjusted post-test thresholds on the AMD 4/2 task for each trained group when tasks were mixed during acquisition and consolidation. The mean pre-test threshold across all participants is provided as a dotted line, with grey filled area denoting the 95% confidence interval. (C) Mean adjusted thresholds on the AMD 8/1 task across each daily session for the secondary task-relevant training group. (D) Mean adjusted post-test thresholds on the AMD 8/1 task for each trained group. (E) Mean adjusted thresholds on the AMR 4/1 task across each daily session for the secondary different task training group. (F) Mean adjusted post-test thresholds on the AMR 4/1 task for each trained group.

Learning of the secondary trained task (Figs. 2 and 3) was observed across all the mixed-training groups (compared to the no-training controls), regardless of whether tasks were interleaved or presented consecutively (Fig. 5). During both acquisition and consolidation, learning curves significantly differed from controls for the secondary task-irrelevant (Fig. 5A; acquisition: *F*(1,27) = 14.87, *p* = .001, *η* ^*2*^_*ρ*_ = .40, α = .008; consolidation: *F*(1,27) = 5.57, *p* = .026, *η* ^*2*^_*ρ*_ = .17, α = .050), the secondary task-relevant (Fig. 5C; acquisition: *F*(1,27) = 8.27, *p* = .008, *η* ^*2*^_*ρ*_ = .24, α = .013; consolidation: *F*(1,27) = 6.32, *p* = .018, *η* ^*2*^_*ρ*_ = .20, α = .025), and the secondary different task training groups (Fig. 5E; acquisition: *F*(1,25) = 7.58, *p* = .011, *η* ^*2*^_*ρ*_ = .23, α = .017; consolidation: *F*(1,25) = 11.14, *p* = .003, *η* ^*2*^_*ρ*_ = .31, α = .010).

Although the acquisition and consolidation learning curves did not significantly differ from each other across any of the secondary tasks (*p* <. 05), it is interesting to note that learning was marginally better for acquisition versus consolidation when the task-irrelevant or task-relevant stimulus feature was varied, but was worse when different tasks (AMD versus AMR) were mixed during training.

### Transfer of learning to AMD tasks, trained stimuli

Transfer of learning to untrained conditions was assessed across all eight groups using separate omnibus ANCOVAs for the post-test thresholds of each task, using pre-test thresholds as the covariate. First, we present the pattern of transfer for tasks on which some of the groups trained in addition to the primary AMD 4/1 task.

#### Transfer to secondary task-irrelevant (AMD 4/2)

In addition to the group that trained on the secondary AMD 4/2 condition, only the group that trained with a mixed task-relevant stimulus (AMD 4/1 and AMD 8/1) showed transfer of learning to the AMD 4/2 condition, and then only when training was mixed during acquisition (Fig. 5B). The ANCOVA on post-test thresholds confirmed a significant effect of group, *F*(7,107) = 2.77, *p* = .011, *η* ^*2*^_*ρ*_ = .15, with pairwise comparisons revealing that that post-test thresholds significantly differed between controls and the secondary task-irrelevant training group that trained on this task during both acquisition (*p* = .002) and consolidation (*p* = .019), as well as the secondary task-relevant group when tasks were interleaved during acquisition (*p* = .006). All other comparisons were not significant.

#### Transfer to secondary task-relevant (AMD 8/1)

Only the groups that trained on AMD 8/1 showed improvement on this task; no transfer was observed in any other group (Fig. 5D). There was a significant effect of group, *F*(7,107) = 4.18, *p* <. 001, *η* ^*2*^_*ρ*_ = .22, but post-test thresholds only differed significantly from controls for the secondary task-relevant group that trained on this task during both acquisition (*p* = .031) and consolidation (*p* = .001).

#### Transfer to secondary different task (AMR 4/1)

Learning only transferred to a different task (AMR) that shared the same stimulus features when the task-irrelevant, but not task-relevant, stimulus feature was mixed during consolidation. Analysis confirmed that there was a significant effect of group for the AMR 4/1 task (Fig. 5F), *F*(7,104) = 2.42, *p* = .024, *η* ^*2*^_*ρ*_ = .14. Pairwise comparisons revealed that post-test thresholds significantly differed from controls for the secondary different task training group (*p* = .003), as well as the secondary task-irrelevant group (*p* = .025) when tasks were mixed during consolidation.

### Transfer to AMD tasks, untrained stimuli

We next examined the transfer of learning to novel AMD conditions that were not trained in any group.

#### Transfer to untrained task-irrelevant feature (carrier frequency; AMD 4/4)

**Fig 6:**
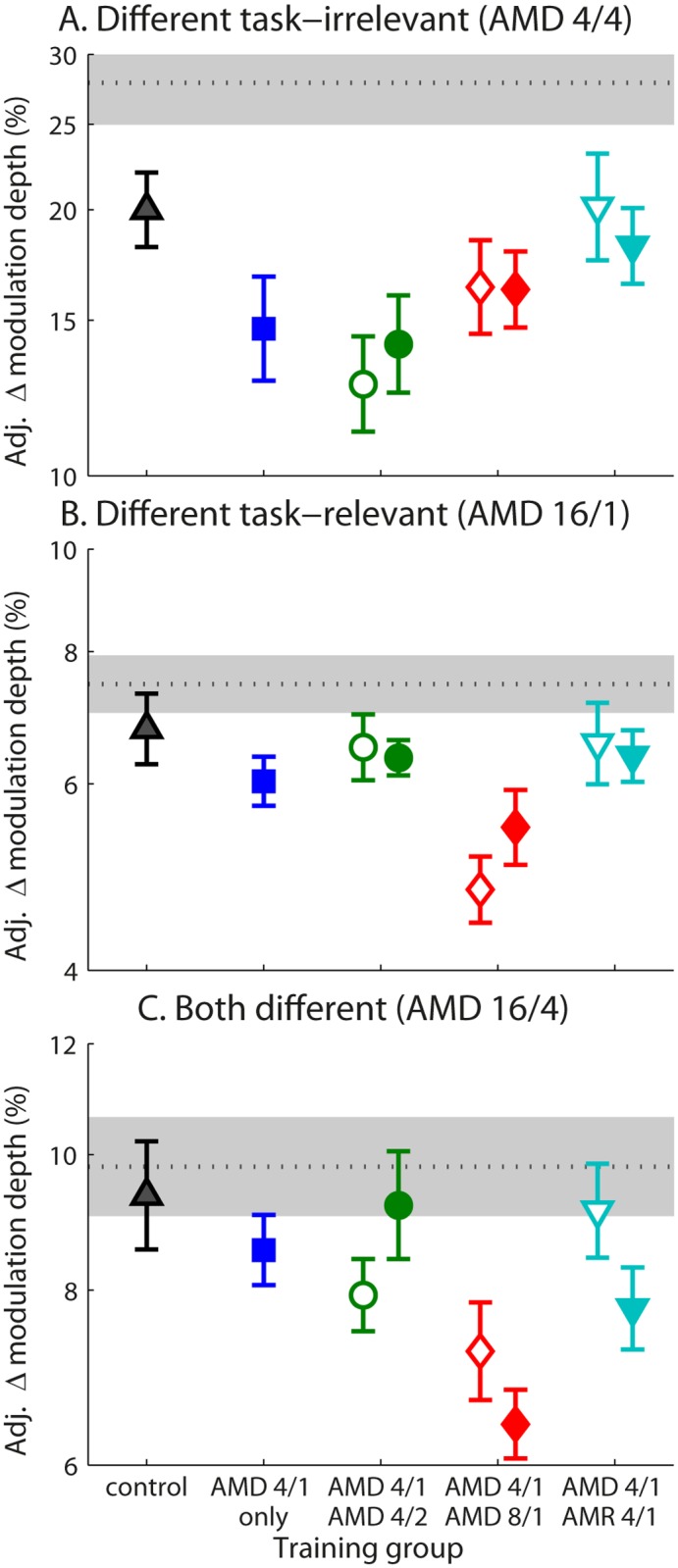
Transfer of learning to untrained AMD conditions. Mean adjusted post-test thresholds on (A) AMD 4/4, (B) AMD16/1, and (C) AMD 16/4 tasks for all groups during acquisition (empty symbols) and consolidation (filled symbols). The mean pre-test threshold across all participants is provided as a dotted line, with grey filled area denoting the 95% confidence interval.

Learning did not transfer significantly for any group to an AMD 4/4 task with an untrained task-irrelevant feature (i.e. carrier frequency) (Fig. 6A). The group effect was not significant, *F*(7,104) = 1.81, *p* = .094, *η* ^*2*^_*ρ*_ = .11.

#### Transfer to untrained task-relevant feature (modulation rate; AMD 16/1)

Mixing the primary AMD 4/1 task with the secondary task-relevant AMD 8/1 task resulted in learning transfer to an AMD with a third, untrained modulation rate (AMD 16/1), irrespective of whether tasks were mixed during acquisition or consolidation (Fig. 6B). There was a significant effect of group for the AMD 16/1 post-test thresholds, *F*(7,107) = 2.72, *p* = .012, *η* ^*2*^_*ρ*_ = .15, with pairwise comparisons revealing significant differences between controls and the secondary task-relevant training group when tasks were mixed during both acquisition (*p* = .001) and consolidation (*p* = .034).

#### Transfer to untrained task-relevant and irrelevant features (AMD 16/4)

Similar to the AMD 16/1 task, training that varied the task-relevant feature (AMD 4/1 and AMD 8/1) transferred to the AMD 16/4 task whether mixed during the acquisition or consolidation phases (Fig. 6C). A significant group effect, *F*(7,105) = 3.14, *p* = .005, *η* ^*2*^_*ρ*_ = .17, followed by pairwise comparisons revealed that the secondary task-relevant training groups differed from controls when tasks were mixed during both acquisition (*p* = .018) and consolidation (*p* = .001).

### Transfer to AMR tasks, trained/untrained stimuli

No transfer was observed by any training group to any of the remaining AMR conditions tested, whether using trained stimuli (AMR 4/2 and 8/1; S1 Fig.) or untrained stimuli (AMR 4/4, 16/1, 16/4; S2 Fig.). All ANCOVAs yielded a non-significant main effect of group (*p* >. 05), suggesting that learning did not transfer to another task requiring a judgment based on a different discrimination cue with either the same or different stimulus features experienced during training.

## Discussion

**Fig 7:**
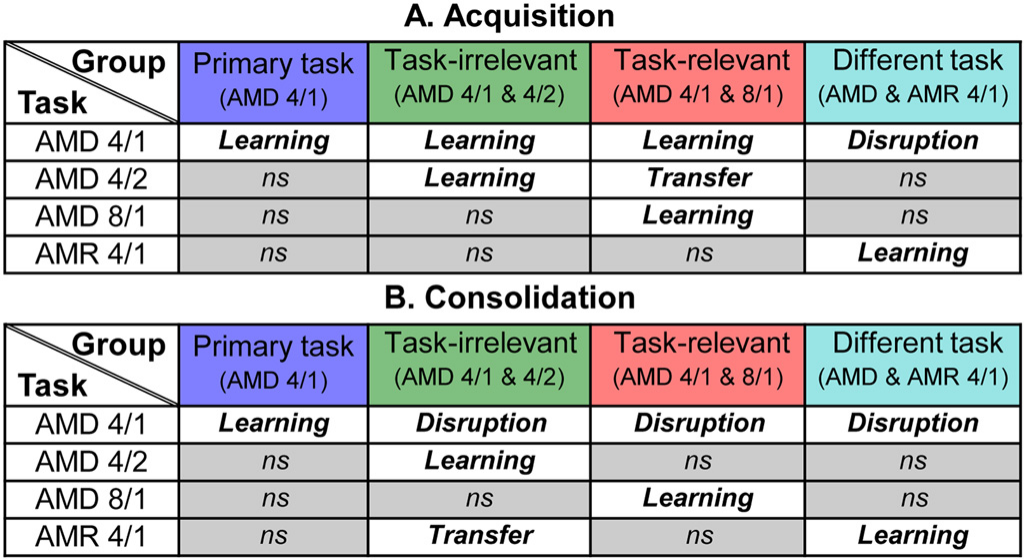
Summary of key results. The pattern of learning, disruption, and transfer observed on the primary and secondary trained tasks during (A) acquisition and (B) consolidation for the primary only, task-irrelevant, task-relevant and different task training groups. Non-significant (ns) results denote that performance did not differ from the no-training control group.

The key findings can be summarized as follows (see also Fig. 7): (1) acquisition of the primary task was disrupted only when two tasks requiring a perceptual judgment based on different cues (modulation depth versus rate) were mixed during training; (2) consolidation of the primary task was always disrupted, at least partially, regardless of whether different stimulus features or tasks were mixed; (3) the secondary task was always learned, regardless of whether the different cues or stimulus features were mixed during acquisition or consolidation; and (4) mixing the task-relevant stimulus feature (during either acquisition or consolidation) resulted in transfer to untrained tasks that differed along the same task-relevant feature, even when the task-irrelevant feature also changed.

While we showed that acquisition and consolidation of a primary AMD task were susceptible to differential patterns of interference, we did not confirm our two predictions regarding what type of change causes interference in each learning phase. First, based on previous literature [4,5,16] we predicted that acquisition would be disrupted by (task-relevant) stimulus inference, but showed instead that it was only disrupted when the trained cue differed between tasks. Second, we expected that consolidation would only be disrupted by changing the perceptual judgment based on different cues between tasks, but found that learning was disrupted by varying either the stimulus (task-irrelevant/relevant) or task (AMD/AMR).

The systematic evaluation of acquisition and consolidation is grounded in the pioneering work of Müller and Pilzecker [29], demonstrating that memory for newly acquired information becomes increasingly resistant to disruption (i.e. learning other information). This led to the proposed consolidation-hypothesis of long-term memory [29], whereby new memories persist in a fragile, transient state, but consolidate over time so that they become robust to subsequent interference, particularly following a period of sleep [15]. The existence of a ‘time-window’ of susceptibility has commonly been used to refer to the consolidation of declarative memory—the conscious awareness of memory retrieval for facts (semantic) or events (episodic) [30,31]. However, the study of implicit, non-declarative (or ‘procedural’) memory, including the study of sensory and motor task learning, suggests that previously consolidated memories are susceptible to competing interference despite undergoing stabilization with or without intervening sleep (e.g. [6,9,32]; for reviews, see [14,33]). Our findings demonstrate that acquisition and consolidation are differentially susceptible to disruption depending on whether stimulus features or the cue are mixed during training.

### Mixing tasks with different stimulus features during training

We found that mixing two task-relevant stimulus features failed to disrupt learning during acquisition but did disrupt consolidation, contrary to a previous study addressing this question. Banai *et al*. [5] found that mixing two auditory duration discrimination tasks of different base durations (the task-relevant feature) during acquisition caused disruption, but presenting them consecutively did not. We propose this discrepancy between studies might reflect the way relevant and irrelevant features of AM and temporal interval stimuli are represented. Whereas temporal information about different durations appears to be encoded in distinct neuronal populations [34], AM-sensitive neurons phase-lock to many different AM rates (including the slower rates deployed in the current study) [35]. We speculate that continuous access to the same neuronal population encoding the relevant stimulus features (as for AM) may not disrupt acquisition, but the cost of switching between different populations (as for duration) may be disruptive. Once consolidation has been initiated, attempting to access a different stimulus representation in the same population may interfere with the consolidation of the primary task representations. However, accessing a different neuronal population by the secondary task cannot interfere with consolidation in a separate population (e.g. encoding a different duration).

This explanation also accounts for the specificity to the task-relevant stimulus feature observed for duration discrimination (see [5]), in comparison to the transfer to an untrained modulation rate observed in the current study (see also [20]). In the former, each new stimulus needs to be trained until it can be consolidated individually, whereas in the latter changing the stimulus disrupts the consolidation of the stimulus-specific representation, facilitating an alternative learning mechanism that is not stimulus specific. This relearning process that occurs during the secondary task training disrupts the consolidation of the primary task. Indeed, when this relearning did not happen (as in the primary only condition), the learning was specific to the trained stimulus features, thus supporting this interpretation.

### Mixing different tasks during training

The current study suggests that perceptual learning is highly specific to the trained cue; mixing two tasks requiring a perceptual judgment based on two different cues but sharing the same stimulus features disrupted learning. The reverse has been shown by previous investigations in both the auditory [4] and visual domains [7]. The key difference between these studies and the current study is that our two tasks share the same task-irrelevant and relevant stimulus features, while these previous studies do not. Modulation rate is relevant regardless of whether the perceptual judgment requires discrimination of depth or rate [36] while carrier frequency is not, whereas frequency is relevant and duration irrelevant for frequency discrimination, and *vice versa* for duration discrimination [4]. Training-related changes at the level of the relevant-feature representation may be overwritten by another task that requires a different judgment based on access to the same neuronal population, resulting in disruption. Two tasks that access different neuronal populations do not interact, and the additional exposure to the irrelevant feature may even enhance the learning associated with that feature [4,7].

### Mixed training in visual learning

Few studies in the visual literature have investigated mixed training, and none of them showed any difference between interleaved and sequential training regimens. For example, Xiao *et al*. [37] and Zhang *et al*. [38] mixed both task and stimulus features (e.g. orientation discrimination at one location and contrast discrimination at another location), but only reported transfer to a third, untrained task that shares different features with the two trained tasks (e.g. orientation with one task and location with the other), giving no information on how the secondary task affected learning of the primary task. Of greater relevance to the current study, Seitz *et al*. [6] showed that training on two different offsets in a hyperacuity task disrupted both acquisition and consolidation of the primary task (learning on the secondary task was not reported). Sequential training on the same two tasks presented at different locations did not disrupt learning of the primary task, nor did interleaved training at two different orientations. Given that neurons in the primary visual cortex (V1) are retinotopically organized for location [39,40] and topographically organized for orientation [41–43], these results support the notion that training two tasks that stimulate the same neuronal population results in disruption to acquisition, while engaging different populations does not. Once acquisition has been compromised, learning cannot consolidate.

Huang *et al*. [11] interleaved training of bisection and Vernier hyperacuity tasks with identical stimuli and found both tasks were learned. This appears to contradict our findings in interleaved AMD and AMR training with the same relevant stimulus features, which resulted in complete disruption to the primary task. Nevertheless, the learning in Huang *et al*.’s [11] experiment was very rapid—no further learning was observed after the first 3 blocks of each task, and there was no evidence of further consolidation. It is quite possible that consolidation on this task requires very few trials, possibly even initiating after a single training block, in which case it is possible that the observed learning is equivalent to the partial learning we observed in the sequential training regimen in the current experiment. We cannot verify this speculation because learning on each individual task is not reported.

It might also be worth pointing out that in the visual learning studies discussed above the distinction between task-relevant and task-irrelevant stimulus features might not be straightforward as it is in the auditory counterpart. This may therefore limit any direct comparison of our findings with those in the visual perceptual learning literature.

### Implications for application

If our findings from AM learning can be generalized to perceptual learning of other tasks, they imply that learning can be optimized (i.e. both tasks are learned) by varying the stimulus during acquisition while keeping to the perceptual judgment (here modulation depth discrimination) consistent. Mixing the task-relevant stimulus feature gives the added advantage that the learning transfers to untrained stimuli. This is only the case if neither trained task is allowed to consolidate. On the other hand, mixing tasks with perceptual judgments based on different cues (modulation depth and rate) may not be suitable during either acquisition or consolidation, with learning only observed for the second trained task, and no transfer to any other untrained conditions.

These findings have potential practical implications for the optimal design and delivery of applied training programs that aim to improve perceptual abilities, though further work would be necessary to extend our findings to real-world tasks. One objective of applied auditory training programs is to improve speech perception in adverse listening conditions (e.g. LACE [44]). Our choice of training AMD and AMR stimuli was geared towards this end because the ability to detect fluctuations in sound amplitude contributes to accurate speech-in-noise perception [45–48]. Moreover, enhancing AMD abilities through training may have significant implications for treatment and intervention strategies that aim to rehabilitate hearing-impaired listeners, particularly those that rely on AM cues (e.g. cochlear implant users) to enhance speech intelligibility [49,50].

To conclude, our findings suggest that mixing tasks during practice is sensitive to the choice of tasks and stimuli. Specifically, optimal learning (in terms of trained tasks as well as transfer to untrained tasks) was achieved by interleaving tasks that required the same judgment but varied the task-relevant stimulus feature in blocks that were short enough to prevent the initiation of consolidation.

## Supporting Information

S1 Fig Transfer of learning to AMR with trained stimuli. Mean adjusted post-test thresholds on (A) AMR 4/2, and (B) AMR8/1 for all groups during acquisition (empty symbols) and consolidation (filled symbols). The mean pre-test threshold across all participants is provided as a dotted line, with grey filled area denoting the 95% confidence intervals. Error bars indicate ±1 standard error of the mean.
(TIF)

S2 Fig Transfer of learning to AMR with untrained stimuli. Mean adjusted post-test thresholds on (A) AMR 4/4, (B) AMR 16/1, and (C) AMR 16/4 for all groups during acquisition (empty symbols) and consolidation (filled symbols). The mean pre-test threshold across all participants is provided as a dotted line, with grey filled area denoting the 95% confidence interval. Error bars indicate ±1 standard error of the mean.
(TIF)
