# Supplementary figures and images for: Acquisition versus Consolidation of Auditory Perceptual Learning Using Mixed-Training Regimens
Source: PLoS One. 2015 Mar 24;10(3):e0121953. doi: 10.1371/journal.pone.0121953 (PMC4372427; doi:10.1371/journal.pone.0121953)

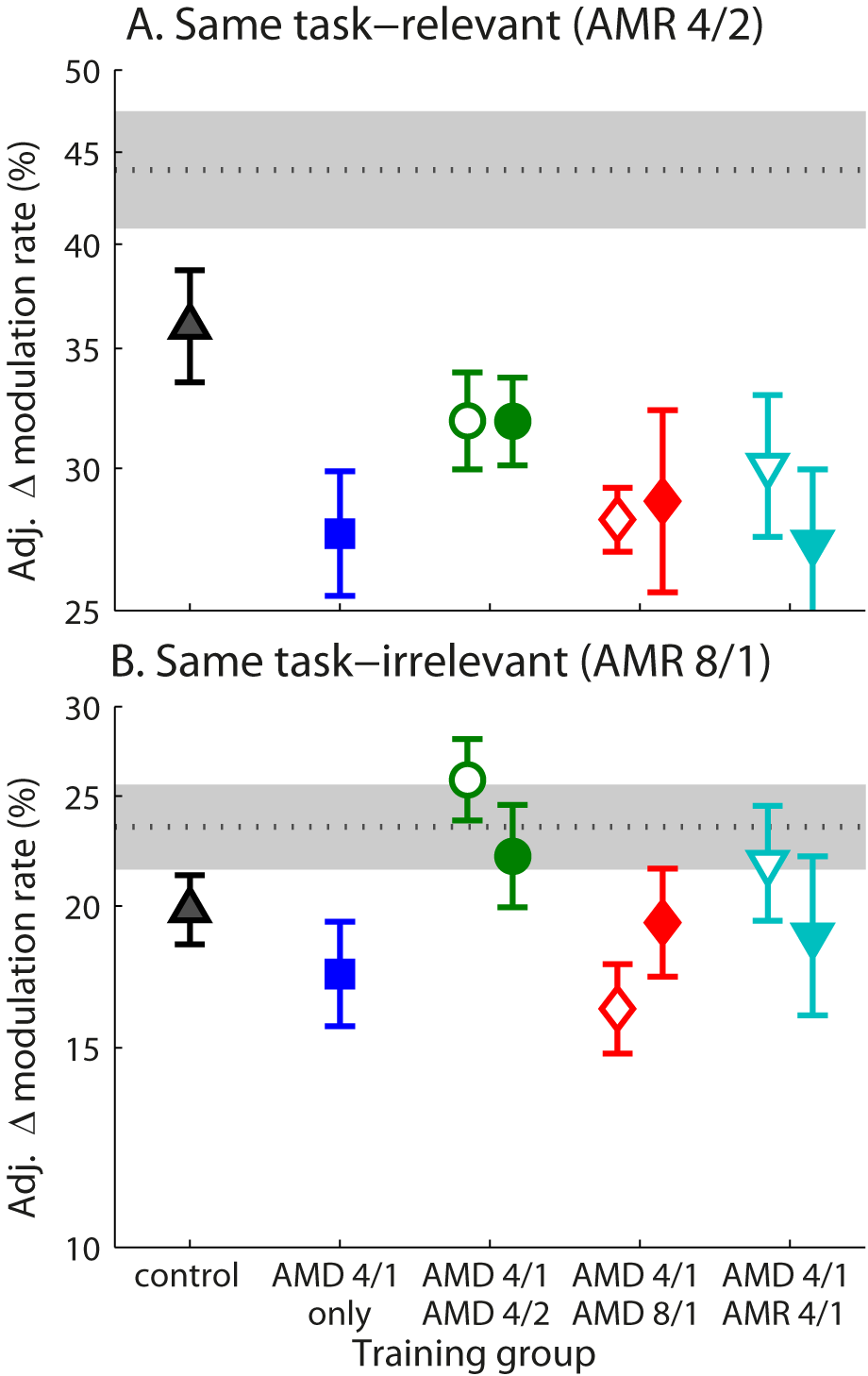

Supplement: S1 Fig — Mean adjusted post-test thresholds on (A) AMR 4/2, and (B) AMR8/1 for all groups during acquisition (empty symbols) and consolidation (filled symbols). The mean pre-test threshold across all participants is provided as a dotted line, with grey filled area denoting the 95% confidence intervals. Error bars indicate ±1 standard error of the mean. (TIF) [file pone.0121953.s001.tif]

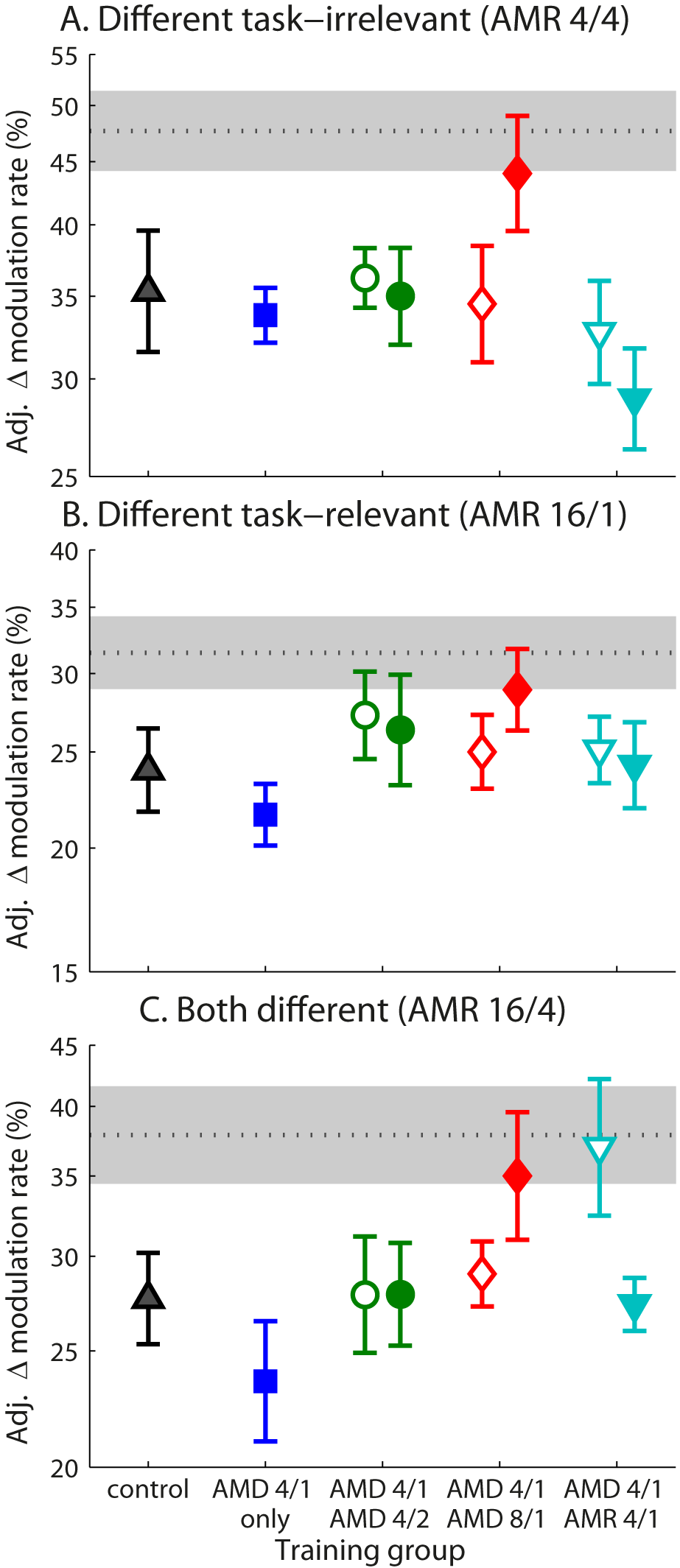

Supplement: S2 Fig — Mean adjusted post-test thresholds on (A) AMR 4/4, (B) AMR 16/1, and (C) AMR 16/4 for all groups during acquisition (empty symbols) and consolidation (filled symbols). The mean pre-test threshold across all participants is provided as a dotted line, with grey filled area denoting the 95% confidence interval. Error bars indicate ±1 standard error of the mean. (TIF) [file pone.0121953.s002.tif]
